# Removal of the 2-Mercaptobenotiazole from Model Wastewater by Ozonation

**DOI:** 10.1155/2014/173010

**Published:** 2014-01-23

**Authors:** Jan Derco, Angelika Kassai, Michal Melicher, Jozef Dudas

**Affiliations:** ^1^Faculty of Chemical and Food Technology, Slovak University of Technology, Radlinského 9, 812 37 Bratislava, Slovakia; ^2^Water Research Institute, Nábr. Arm. Gen. L. Svobodu 5, 812 49 Bratislava, Slovakia

## Abstract

The feasibility of ozonation process for 2-mercaptobenzothiazole (2-MBT) removal follows from results of ozonation of the model wastewater. Total removal of 2-MBT was observed after 20 minutes of ozonation. Very good reproducibility of repeated ozonation trials including sampling and analysis was observed. However, the majority of dissolved organic carbon (DOC) and chemical oxygen demand (COD) remained in the reaction mixture. Benzothiazole (BT) and 2-hydroxybenzothiazole (OBT) intermediates were identified during degradation of 2-MBT with ozone. In addition to the above benzothiazole derivatives, the creation of some other organic compounds follows from results of mass balance. The best fits of experimental data were obtained using the first kinetic model for 2-MBT and zero-order kinetic model for COD and DOC. The reaction time of 60 minutes can be considered as effective with regard to controlled oxidation in order to increase a portion of partially oxidized substances. Higher biodegradability and lower toxicity of ozonation products on respiration activity of activated sludge microorganisms was observed at higher ozonation time.

## 1. Introduction

Ozone is very strong oxidant and reacts with most of inorganic and organic pollutants. It is used in water and wastewater treatment. Ozone can react directly with a compound or trough reactions of hydroxyl radicals generated in the ozone decomposition that then react with a compound. It appears as an attractive treatment option due to its well-known capacity to oxidise aromatic compounds [1].

Benzothiazoles are toxic and poorly biodegradable pollutants. Benzothiazole and its derivatives are widely used as industrial chemicals in the leather and wood industries, as biocorrosion inhibitors in cooling systems, ingredients in antifreezing agents for automobiles, and mainly as vulcanization accelerators in rubber production. They are often used, as herbicides and fungicides, as anti-fungal drug, as corrosion inhibitors in cooling water, as slimicides in the paper and pulp industry, and mainly as vulcanization accelerators in rubber production [2]. Correspondingly, these xenobiotic compounds are widely distributed in the environment and have been detected in industrial wastewater, as well as in soils, estuarine sediments, and superficial water [3]. Benzothiazole compounds have been detected in various environmental compartments: in wastewaters, soils, estuarine sediments, and superficial waters [2].

Benzothiazoles pose an environmental concern when released into watercourses [4]. These compounds inhibit microorganisms activity in conventional biological wastewater treatment systems and most of them are not readily biodegradable [2, 5]. Moreover, these compounds can be absorbed into cell membranes, leading to bioaccumulation [2]. Unfortunately, conventional biological wastewater treatment cannot effectively remove such contaminants, since they are resistant to biodegradation [2].

2-Mercaptobenzothiazole (2-MBT) is widespread toxic and poorly biodegradable substance. Moreover, 2-MBT inhibits degradation of easily degradable organics and wastewater nitrification is inhibited at far lower concentrations [6]. This compound is known to be toxic to aquatic organisms [7], allergenic [8], and inducing tumours [9]. Published data related to biodegradation of MBT are ambiguous. Mainprize et al. [10] and Gaja and Knapp [11] reported that it may be metabolised (although incompletely) by microorganisms which have been cultivated on other benzothiazoles. Chudoba et al. [12] suggested MBT as a recalcitrant to biodegradation. The removal of MBT by activated sludge is specified as a nonenzymatic process [11]. It may also hamper wastewater treatment processes at concentrations higher than 600 *μ*mol·L^−1^ [13]. Fiehn et al. [6] have found MBT to inhibit degradation of easily degradable organics and the process of nitrification was inhibited at a far lower concentration. MBT is readily transformed to oxidation products by ozonation and wastewaters containing MBT should easily be detoxified using ozone [6]. Thus, the development of efficient treatment/pretreatment processes is required in order to eliminate their discharge into aquatic ecosystems.

Puig et al. [14] studied the ozonation of complex wastewater containing eight benzothiazole derivatives. They achieved complete removal of 2-MBT but did not detect any benzothiazole derivative although there was no decrease in dissolved organic carbon (DOC). Fiehn et al. [6] investigated the ozonation of model wastewater containing 600 *μ*mol^−1^ of 2-MBT at pH 3, 7, and 9. All tests were carried out at a constant pH. An aerobically treated tan-yard effluent was taken for a comparative ozonation of 2-MBT at pH 7. This wastewater sample was also spiked with 600 *μ*mol^−1^ of 2-MBT. During ozonation maximum value of BT, OBT, and BTSO2 was measured after 6, 12.5, and 15 minutes and the process took over 55 minutes to remove all identified benzothiazole derivatives, including 2-MBT.

No significant differences in the results of ozonation carried out at the above pH values were observed. During the comparative ozonation with aerobically treated tan-yard effluent spiked with the same amount of 2-MBT, the same intermediates were observed at the same time periods. For BT the same concentration was measured as during ozonation of the model wastewater. OBT concentration reached 1.5 times of the maximum concentration and BTSO2 was about half compared with the measured values in the ozonation of the model wastewater. Based on the total balance of dissolved organic carbon (DOC), however, the authors stated that at the end of the process about 75% of the residual DOC remained unidentified. Therefore, benzothiazole derivatives may still enter the aqumineatic environment, if the 2-MBT-containing effluents are not biologically degraded with suitable bacterial sludge after the ozonation [6].

This work aims to study the kinetics of degradation and transformation of 2-MBT with ozone and monitor the impact of ozonation products on activated sludge microorganisms. The aim is to find conditions for controlled ozonation 2-MBT in terms of its partial transformation, complete mineralization, and reduce toxicity to activated sludge microorganisms. Another objective is to determine the specific parameter values for 2-MBT and verify the reproducibility of experimental measurements including analytical determination of indicators 2-MBT, COD, and DOC.

## 2. Materials and Methods

### 2.1. Characterisation of Wastewater

The wastewater from rubber industry contains more than 200 mg·L^−1^ of 2-MBT [1, 15]. Similar content of 2-MBT was measured in the wastewater produced from N-cyclohexyl-2-benzothiazol-sulfenamide [16, 17]. The concentration of MBT in model wastewater varied from 661 to 1055 mg L^−1^ for this set of experiments. The chemical was provided by Merck (≥95.5% purity).

### 2.2. Experimental Equipment and Procedures

**Figure 1:**
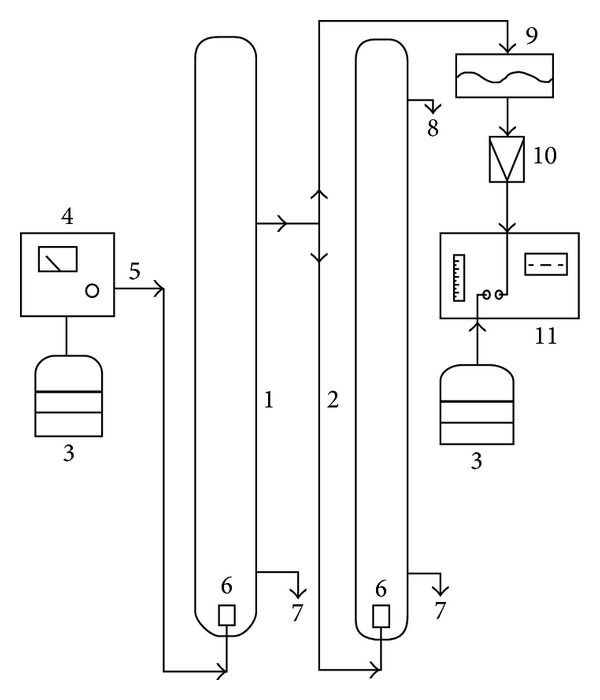
Schematic diagram of experimental apparatus. 1: ozonation column, 2: destruction of residual ozone, 3: oxygen supply, 4: ozone generator, 5: mixture of oxygen and ozone, 6: ozone distribution, 7: sampling, 8: residual gas outlet, 9: separation of moisture, 10: glass fiber filter, 11: UV analyzer of ozone content in gas phase.

Ozonation experiments were carried out in two glass columns, 0.04 m diameter and 1.70 m height. The first column was filled with activated sludge sample, and the other one with solution of potassium iodide. The reason was to specify the amount of residual ozone as well as to destroy residual ozone in the outlet of ozonation column. The effective volume of both columns was 1.0 litre. Schematic diagram of experimental ozonation apparatus is shown in Figure 1. The Lifetech ozone generator with the maximum ozone production 5.0 g·h^−1^ was used. Mixture of ozone and oxygen was introduced into ozonation column at the bottom, and it was mixed with model wastewater sample through fine-bubble porous aeration element.

The system was operated in batch mode with regard to wastewater samples. Wastewater samples were added to ozonation column at the beginning of trials. Continuous flows of oxygen at 20 L·h^−1^ (*T* = 22.0°C, *p* = 99 800 Pa) were applied for generation of ozone. Ozonation trials were carried out at performance of ozone generator 70% of the power maximum. Ozonation times were from 20 to 60 minutes. At selected time intervals, samples were collected and analyzed for indicators COD and identified benzothiazoles (BT, 2-MBT, OBT).

The outlet gas mixture was conducted into second bubble column through a fine-bubble porous distribution element. The dimensions of the column were identical to ozonation column. The column was filled with a solution of potassium iodide. The excess ozone destruction was carried out in this column. Likewise as the ozonation column, the effective volume of the bubble column was 1.0 L. The concentration of ozone in gas phase was measured using Life ODU 200 Analyzer.

The influence of raw and ozonated wastewater samples on activated sludge microorganisms activity was evaluated based on results obtained by respirometric measurements. Oxygen uptake rates by microorganisms of activated sludge, which was cultivated in semicontinuous laboratory scale activated sludge process were measured [18].

### 2.3. Analytical Procedures

2-MBT and other BT derivatives were analysed using Hewlett Packard Liquid Chromatography series II 1090 with DAD detector. Direct injection method was applied using linear gradient of RP-HPLC with UV-DAD detector on the column C18 (Merck). TOC was measured with analyzer Shimadzu TOC-V_CPH/CPN_ (Japan). Analytical control of model wastewater during the treatment procedures included also COD (chemical oxygen demand) determination [19, 20].

### 2.4. Mathematical Treatment of Experimental Data

Experimental data were fitted by zero (see (1), first see (2) and then see (3)) order reaction kinetic models. For a batch reaction system, under the assumption of a constant reaction volume, the following relationships are obtained

(1) $$\begin{array}{c} \text{CO}{\text{D}}_{t}=\text{CO}{\text{D}}_{0}-k_{0}t, \end{array}$$

(2) $$\begin{array}{c} \text{CO}{\text{D}}_{t}=\text{CO}{\text{D}}_{0}\exp\left(-k_{1}t\right), \end{array}$$

(3) $$\begin{array}{c} \text{CO}{\text{D}}_{t}=\frac{\text{CO}{\text{D}}_{0}}{\left(1+\text{CO}{\text{D}}_{0}k_{2}t\right)}, \end{array}$$

where COD_*t*_/(g m^−3^) denotes the value of COD in wastewater in time *t*, COD_0_/(g m^−3^) the initial value of COD in wastewater, and *k*_0_/(g m^−3^ h^−1^), *k*_1_/h^−1^, *k*_2_/(g^−1 ^m^3 ^h^−1^) the rate constants for the kinetics of zero, the first, and the second order, respectively.

The parameters of the applied kinetic models were calculated by the grid search optimization procedure. The residual sum of squares (*S*_*r*_^2^) between the observed values and the values given by the model divided by its number of degrees of freedom *ν* (the number of observations less the number of parameters estimated) was used as the objective function.

### 2.5. Partial Oxidation and Mineralization

To evaluate the oxidized and mineralized portions of synthetic sewage pollution during ozonation and identification of the highest efficiency of partial oxidation, we used following equation [21, 22]:

(4) $$\begin{array}{c} \alpha {\text{COD}}_{\text{oxi}}=1-\frac{{\text{COD}}_{t}}{{\text{COD}}_{0}}, \end{array}$$

(5) $$\begin{array}{c} \alpha {\text{COD}}_{\text{miner}}=1-\frac{{\text{DOC}}_{t}}{{\text{DOC}}_{0}}, \end{array}$$

(6) $$\begin{array}{c} \alpha {\text{COD}}_{\text{partoxi}}=\alpha {\text{COD}}_{\text{oxi}}-\alpha {\text{COD}}_{\text{miner}}, \end{array}$$

(7) $$\begin{array}{c} \mu {\text{COD}}_{\text{partoxi}}=\frac{\alpha {\text{COD}}_{\text{partoxi}}}{\alpha {\text{COD}}_{\text{oxi}}}, \end{array}$$

where *α*COD_oxi_ is the proportion of oxidized organic pollutants expressed as COD, *α*COD_miner_ is the proportion of oxidized COD, which was completely mineralized, and *α*COD_partoxi_ is only partially oxidized COD of the synthetic wastewater during the process. The effectiveness of partial oxidation *μ*COD_partoxi_ is proportional to the ratio of decrease in COD due to the transformation of substances intermediates (*α*COD_partoxi_) and the decrease of oxidized COD of the synthetic wastewater (*α*COD_oxi_) in the process.

### 2.6. Impact on Activated Sludge Microorganisms

Respirometric measurements [22] were conducted to assess the impact of ozonation products on activated sludge microorganisms. The measurement results have been processed using the following Monod equation (see (8), [22], and for Haldan equation see (9)) [23, 24]:

(8) $$\begin{array}{c} r_{X}=r_{X,\max}\frac{S}{K_{S}+S}, \end{array}$$

(9) $$\begin{array}{c} r_{X}=r_{X,\max}\frac{S}{K_{S}+S+\left(S^{2}/K_{I}\right)}, \end{array}$$

where *r*_*X*_ and *r*_*X*,max⁡_ are, respectively, the specific respiration rate and maximum specific respiration rate [mg·g^−1^·h^−1^], and *S*, *K*_*S*_, and *K*_*I*_ are the substrate concentration, half-saturation constant, and inhibition constant [mg·L^−1^]. The values of kinetic parameters in (8), and (9) were determined using the grid search method.

### 2.7. Mean Oxidation State of Organic Carbon

Wastewater treatment processes try to convert the environmentally harmful carbon species into a less problematic one, often CO_2_. A suitable method to monitor the fate and oxidation state of the total organically bound carbon is the mean oxidation number of organic carbon (MOC) as proposed by Stum and Morgan [25] and extended by Vogel et al. [26]. For wastewater containing more pollutants, COD and TOC values can be combined to give a MOC estimate according to (10):

(10) $$\begin{array}{c} \text{MOC}=4-1.5\frac{\text{CO}{\text{D}}_{\text{o}}}{\text{TOC}}, \end{array}$$

where COD_o_ is the organic COD and TOC is total organic carbon.

## 3. Results and Discussion

Three independent series of measurements, with reaction times of 20–60 minutes, were performed. Thus, overall 23 experimental values were used for each monitored variable to evaluate the kinetics. The pH values varied in the range from 12.3 to 7.9 during ozonation experiments.

**Figure 2:**
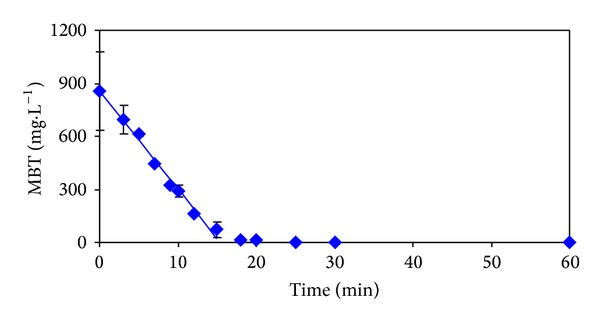
Dependencies of measured (◆) and calculated (—) 2-MBT values on ozonation time.

**Figure 3:**
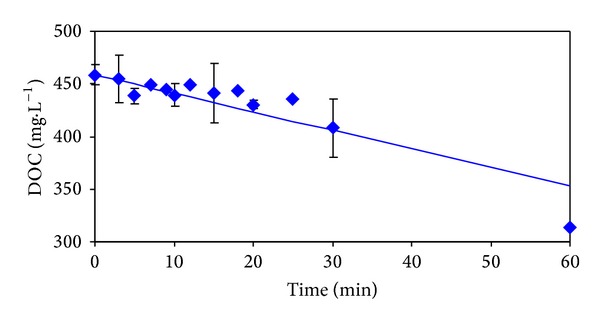
Dependencies of measured (◆) and calculated (—) DOC values on ozonation time.

**Figure 4:**
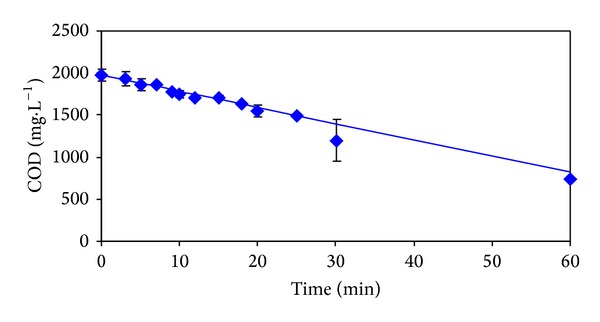
Dependencies of measured (◆) and calculated (—) COD values on ozonation time.

The time dependencies of measured and calculated values of 2-MBT, COD, and DOC are presented in Figures 2 to 4. The boundaries of the 95% confidence intervals, which were calculated on the basis of statistical analysis of repeated measurements, are also marked in these figures.

There can be seen two regions in Figure 2. During the first region with duration of approximately 15 minutes, the conversion of 2-MBT to BT and other BT-derivatives occurs according to mechanisms proposed by Fiehn et al. [6] and results published by Derco et al. [17, 27]. Kinetic data measured for this region were fitted very well by zero-order kinetics. The trend of 2-MBT transformation is very similar with the trends of DOC and COD degradation kinetics (Figures 3 and 4). However, from the values of zero-order kinetic constants (Table 1) it follows that transformation and degradation processes occur simultaneously with obviously higher transformation rate of 2-MBT in comparison to degradation rates of DOC and COD. Relatively very low 2-MBT concentration values and consequently also 2-MBT decline rates are obvious for the second stage of 2-MBT dependence on ozonation time.

From Figure 2 it is evident that during the first 20 minutes of the process performance almost complete elimination of 2-MBT in the model wastewater occurred. However, after this reaction time, 93.8% of the original value of DOC (Figure 3), and 75.5% of the original value of COD (Figure 4) remained in the model wastewater.

It is obvious that 2-MBT was only partially oxidized and majority was transformed to intermediates and products, respectively simple benzothiazole derivatives, which remained in the treated model wastewater. During the next 40 minutes of ozonation performance DOC value declined to 68.5% and COD decreased to 37.6% of the initial values.

Calculated values in Figures 2 to 4 correspond to the best descriptions of the experimental values using conventional kinetic models. The values of kinetic parameters and statistical characteristics (correlation coefficient and residual dispersion) are for individual indicators of pollution and kinetic models with the best description of the experimental values given in Table 1. The best description of the experimental values of DOC and COD was achieved using zero kinetics (Table 1). Removal of 2-MBT corresponds to the first kinetic model (Table 1).

From dependencies pictured in Figures 2 to 4 from the values of the statistical characteristics given in Table 1, and it can be seen that the largest residual dispersion between measured and calculated values shows time dependence of 2-MBT values. In Table 2 there are given the results of statistical treatment of repeated experiments which were carried out to verify the reproducibility of the results achieved. The removals of studied pollution indicators depending on reaction time are graphically illustrated in Figures 2 to 4.

The results in Table 2 show the largest variability of COD values measured after 15 minutes of ozonation. In the case of 2-MBT, the value of 95% confidence interval did not exceed 3% of the average 2-MBT value after 15 minutes of ozonation. The accuracy of repeated measurements for DOC and COD pollution indicators increases at longer reaction times. The limit values for 95% confidence intervals for these indicators of pollution did not exceed 3% of the average values after 60 minutes of ozonation. It can be concluded that the reproducibility of the results of repeated ozonation assays, including sampling and analysis, was very good.

From the statistical analysis of repeated measurements of initial values, the following specific values for 2-MBT have been obtained: COD/2-MBT = 2.008 g·g^−1^ (limit value for % confidence interval is ±0.145 g·g^−1^) and DOC/2-MBT = 0.463 g·g^−1^ (limit value for 95% confidence interval is ±0.032 g·g^−1^).

**Figure 5:**
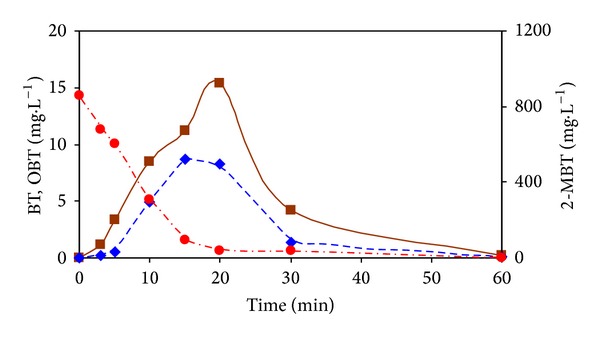
Dependence of concentrations (●) 2-MBT, (◆) BT, and (■) OBT on ozonation time.

In Figure 5 are in addition to 2-MBT shown also time dependencies of identified intermediates (BT and OBT) of 2-MBT transformation with ozone. It can be seen from these dependencies that to a maximum decrease of 2-MBT concentration correspond maximum values of the degradation products. After removal/transformation of 2-MBT the content of these derivatives gradually decreases to zero concentration.

Taking into account the above values of DOC and COD, it is obvious that in the sample of ozonated model wastewater there are accumulated so far unidentified reaction products.

**Figure 6:**
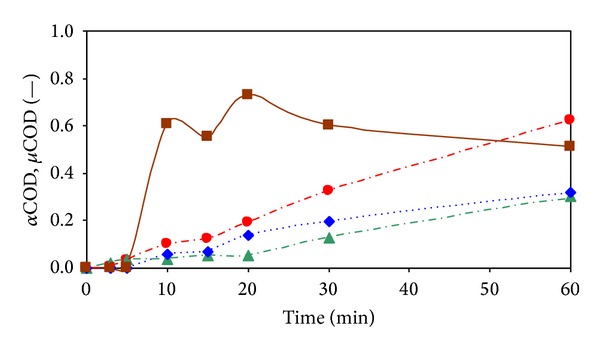
Dependencies of COD portions on ozonation time. (●) *α*COD_oxi_, (▲) *α*COD_min⁡_, (◆) *α*COD_parcoxi_, and (■) *μ*COD_partoxi_.

Figure 6 shows the dependencies of oxidation, mineralization, partial oxidation portions, and the effectiveness of partial oxidation (see (4)–(7)) on ozonation time during 60 minutes oxidation assay with the model sample of 2-MBT. Of these dependencies it follows that time courses of the portions of COD oxidation (see (4), (*α*COD_oxi_)), mineralization (see (5), (*α*COD_min⁡_)), and partial oxidation (see (6), (*α*COD_partoxi_)) exhibit an approximately linear dependence on the reaction time. High partial oxidation efficiency (see (7), *μ*COD_partoxi_ about 73%) corresponds to the reaction time of 20 minutes. Due to the fact that this response time corresponds to a relatively low proportion of *α*COD_oxi_ (19.3%), of which the portion of *α*COD_partoxi_ is 14.1%, these parameters cannot be considered to be effective in terms of controlled oxidation in order to increase the portion of partially oxidized substances with higher oxygen content prior to completing mineralization processes by economically the most convenient biological wastewater treatment processes. From this aspect an effective reaction time is 60 minutes (*α*COD_oxi_ = 62.3%), while the proportion of partially mineralized *α*COD_partoxi_ is 51.0%.

**Figure 7:**
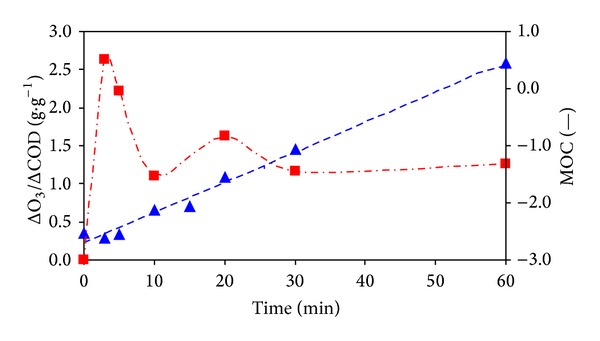
Dependencies of ΔO_3_/ΔCOD (■) and MOC (▲) values on ozonation time.

This is confirmed by the time dependencies of specific consumption of ozone related to unit mass of COD reduction (ΔO_3_/ΔCOD) and the mean oxidation number of organic carbon MOC [22, 26], which are shown in Figure 7.

**Figure 8:**
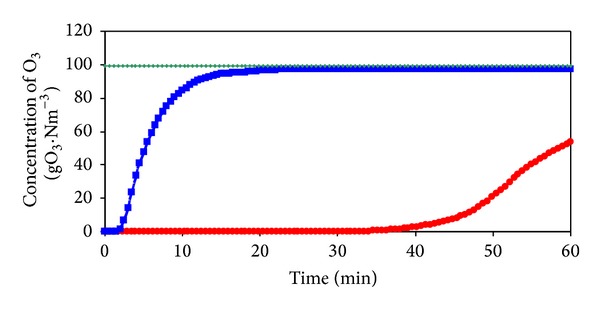
Time dependencies of O_3_ concentration in the gaseous phase. — Input, ● Output 2-MBT, and ■ Output H_2_O.

In Figure 8 the time courses of ozone concentrations are shown measured under the same conditions at the inlet and outlet of the ozonation column filled both, with demineralized water and the model wastewater. It can be seen that, during approximately 40 minutes of ozonation of the model wastewater, nearly all supplied ozone was transferred to the liquid phase. After this time, ozone concentration in the gas phase gradually increased, indicating a less consumption of ozone for oxidation of created intermediates and products. This indicates also the reaction conditions, at which the ozonation process is not limited by the amount of ozone supplied. Based on ozone balance can be stated that 98.6% of O_3_ was transferred to liquid phase.

**Figure 9:**
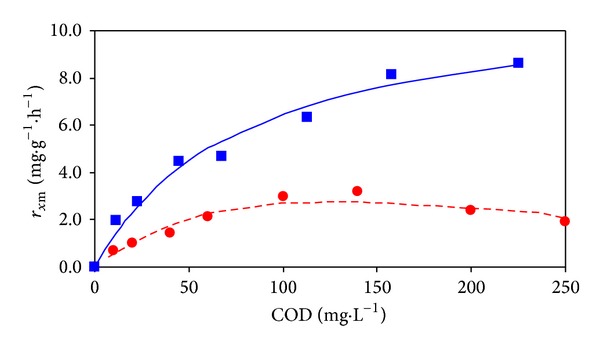
Dependencies of SOUR with the activated sludge microorganisms on COD values during respirometric measurements performed with ozonized model wastewater containing 2-MBT during 15 minutes (●) experimental, (- - -) calculated and during 40 minutes (■) experimental, (—) calculated.

The results of respirometric measurements to assess the impact of products generated during the ozonation of the model wastewater containing 2-MBT on the activity of the activated sludge microorganisms are shown in Figure 9. Respirometric measurements were performed with activated sludge, which was cultivated in semicontinuous lab-scale model of activated sludge process (glucose, peptone, sludge age 10 days). The first ozonation of the model wastewater lasted 15 minutes. From the dependence of specific oxygen uptake rate on COD value it is clear that at higher COD values there exists inhibition of respiratory activity of activated sludge microorganisms. For a description of this dependence Haldane kinetic model was used (see (9)). The second curve in Figure 9 corresponds to the measurement of respiratory activity after the addition of ozonized model wastewater samples containing 2-MBT after 40 minutes ozonation. For a description of this dependence Monod kinetic model was used (see (8)). Parameter values of Monod and Haldane equations and correlation coefficient values are given in Table 3. The maximum values of specific respiration rate obtained for ozonized samples of synthetic sewage of 2-MBT are significantly smaller than for reference sample glucose (*r*_*x*,*m*_ = 99.5 mg·g^−1^·h^−1^). On the other hand, respirometric measurements indicate that the inhibitory effect of a model substance and its degradation products to ozone can be reduced by a longer duration of the ozonation.

## 4. Conclusions

From the evaluation of the results of repeated measurements it can be concluded that the reproducibility of treatment assays including sampling and analysis was very good. The best description of the experimental 2-MBT, COD, and DOC data was obtained by using zero kinetic model. Almost complete removal of 2-MBT was observed after 20 minutes of ozonation. However, in the reaction mixture there were 93.8% of DOC and 75.5% of COD of the original values at that time. These results confirm that at the first stage of 2-MBT oxidation with ozone prevails its transformation into other organic materials. Benzothiazole derivatives OBT and BT were identified as emerging intermediates during decomposition of 2-MBT with ozone. Results of mass balance of carbon indicate that also other organic compounds are created. The reaction time of 60 minutes with 51.0% proportion of partially oxidized COD was found to be effective in terms of controlled oxidation in order to increase the amount of partially oxidized compounds. From the balance of ozone amounts at the inlet and outlet of the reactor it follows that 98.6% of supplied ozone during the ozonation was transferred from gas into liquid phase. The results of respirometric measurements show that the specific respiration rate of the model wastewater sample ozonated for 40 minutes was significantly higher compared to the sample after 15 minutes of ozonation. This can be explained by the fact that the sample ozonated for 15 minutes contained more less ozonated substances, which are toxic on microorganisms of activated sludge at higher concentrations, reducing their respiration activity and having an inhibitory effect.

## Figures and Tables

**Table 1:** The values of the kinetic parameters and statistical characteristics.

Variable	*N*	*k*_*n*_	Dimension of *k*_*n*_	*r*_*XY*_^2^	*S*_*r*_^2^
MBT	0	55.59	mg·L^−1^ min^−1^	0.9888	968
DOC	0	1.77	mg·L^−1^ min^−1^	0.7590	248
COD	0	19.21	mg·L^−1^ min^−1^	0.9175	8143

**Table 2:** Results of statistical processing achieved removal efficiencies % in repeated experiments aimed to verify the reproducibility of results.

Reaction time	15 min		30 min		60 min	
Variable	Pollutant average efficiency	95% confidence interval	Pollutant average efficiency	95% confidence interval	Pollutant average efficiency	95% confidence interval
2-MBT	97.2	2.9	—	—	—	—
DOC	4.6	1.0	15.5	1.3	36.5	0.8
COD	27.1	9.6	41.3	2.3	65.8	1.8

**Table 3:** Kinetic parameters and correlation coefficients.

Kinetic equation	r_x,m_ (mg·g^−1^·h^−1^)	K_S_ (mg·dm^−3^)	K_I_ (mg·dm^−3^)	r_xy_^2^ (—)
Monod	11.6	70.2	—	0.9767
Haldane	18.1	334.4	44.1	0.9275
